# Housing First Improves Adherence to Antipsychotic Medication Among Formerly Homeless Adults With Schizophrenia: Results of a Randomized Controlled Trial

**DOI:** 10.1093/schbul/sbw136

**Published:** 2016-09-24

**Authors:** Stefanie N. Rezansoff, Akm Moniruzzaman, Seena Fazel, Lawrence McCandless, Ric Procyshyn, Julian M. Somers

**Affiliations:** 1 Somers Research Group, Faculty of Health Sciences, Simon Fraser University, Burnaby, BC, Canada;; 2 Department of Psychiatry, Warneford Hospital, University of Oxford, Oxford, UK;; 3 Faculty of Health Sciences, Simon Fraser University, Burnaby, BC, Canada;; 4 Department of Psychiatry, University of British Columbia, Vancouver, BC, Canada

**Keywords:** homelessness, medication possession ratio, Assertive Community Treatment

## Abstract

Adherence to antipsychotic medication is a significant challenge among homeless patients. No experimental trials have investigated the impact of Housing First on adherence among patients with schizophrenia. We investigated whether Housing First in congregate and scattered-site configurations resulted in superior adherence compared to usual care. Adult participants (*n* = 165) met criteria for homelessness, schizophrenia, and initiation of antipsychotic pharmacotherapy prior to recruitment to an unblinded, 3-arm randomized controlled trial in Vancouver, Canada. Randomization arms were: congregate Housing First (CHF) with on-site supports (including physician and pharmacy services); scattered-site Housing First (SHF) with Assertive Community Treatment; or treatment as usual (TAU) consisting of existing services. Participants were followed for an average of 2.6 years. Adherence to antipsychotic medication was measured using the medication possession ratio (MPR), and 1-way ANOVA was used to compare outcomes between the 3 conditions. Data were drawn from comprehensive pharmacy records. Prior to randomization, mean MPR among participants was very low (0.44–0.48). Mean MPR in the follow-up period was significantly different between study arms (*P* < .001) and approached the guideline threshold of 0.80 in SHF. Compared to TAU, antipsychotic adherence was significantly higher in SHF but not in CHF. The results demonstrate that further implementation of SHF is indicated among homeless people with schizophrenia, and that urgent action is needed to address very low levels of antipsychotic adherence in this population (trial registration: ISRCTN57595077).

## Introduction

Serving homeless people with psychiatric disorders is a complex and global challenge. Reports released prior to the current refugee crisis estimated that more than 400000 people in the European Union were homeless on any given night,^1^ and another 700000 in the United States and Canada.^2,3^ The prevalence of schizophrenia among the homeless has been estimated at approximately 11%.^4^

Poor adherence to antipsychotic medication is a significant problem among homeless people,^5,6^ leading to increased risk of relapse, hospitalization and suicide,^7^ arrest, violence and victimization,^8^ and greater overall public costs.^9^ Duration of homelessness is significantly associated with suboptimal adherence to prescribed antipsychotic regimens.^10^ Current antipsychotic treatment protocols provide little guidance on how to care for individuals who are concurrently homeless and seriously mentally ill.

Housing First,^11^ a housing-based intervention implemented throughout the United States, Canada, and several European countries, prioritizes rapid, low-barrier (nonabstinence based) re-housing for individuals who are chronically homeless and who experience serious psychiatric illness (eg, schizophrenia), problematic substance use, and/or compromised physical health. Support services are delivered using a case management model that tailors services to individual client needs. Housing First has been implemented using both “scattered-site” market housing^11^ (where clients are offered a choice of individual market rental units) as well as “single-site” or congregate configurations^12^ (where clients reside in separate units within a single housing project).

Some evidence suggests that Assertive Community Treatment (ACT)—the case management model integral to scattered-site Housing First (SHF)—may be effective at increasing adherence to antipsychotics among homeless and precariously housed individuals.^13–15^ Moreover, experimental research on congregate-style supportive housing (with on-site supports) found significant improvement in clinical symptoms among severely mentally ill people at high risk of homelessness compared against community market housing with mobile ACT supports.^16^ The authors suggested that this outcome may have been attributable to improvements in medication adherence, although medication use was not measured directly.

No randomized controlled trial has evaluated the impact of either congregate Housing First (CHF) or SHF on adherence to antipsychotics among formerly homeless adults with psychotic disorders. The current analysis used comprehensive administrative data and a randomized controlled design in the context of comprehensive prescription drug coverage to investigate the impact of both these models on antipsychotic medication adherence compared to treatment as usual (TAU) among homeless adults diagnosed with schizophrenia. We used a 3-arm design, comprised of SHF with ACT, CHF with on-site supports, and TAU. We hypothesized that both formats of Housing First would be associated with significantly higher rates of adherence to antipsychotic medication than TAU.

## Methods

### Study Design and Participants

This study was reviewed and approved by the Research Ethics Board at Simon Fraser University. Participants were enrolled in the Vancouver At Home study: Housing First plus ACT vs CHF plus supports vs TAU (ISRCTN57595077)—a 3-arm, randomized controlled trial involving homeless mentally ill adults. Randomization was performed using a centralized computer-generated procedure. Interviewers uploaded data on laptop computers with live connections and received randomization results. Following baseline interviews, participants randomized to SHF or CHF were immediately directed to appropriate service representatives. Further details of the Vancouver At Home protocol, including measures and procedures not included in this study, have been published separately.^17^

For the purposes of the current study, participants were asked to provide separate written informed consent for researchers to receive administrative data, including prescription details. A baseline interview collected self-reported participant history and sociodemographic details (eg, gender, ethnicity, health status, duration of homelessness, etc.). The Multnomah Community Ability Scale^18^ and the SF-12 Health Survey^19^ were also administered to assess illness severity and functional health and well-being. Participants received a stipend ($25.00) for the baseline interview.

### Recruitment and Eligibility

Recruitment involved community agencies serving homeless and mentally ill individuals in Vancouver. Eligible participants were Canadian citizens (eligibility for public benefits received by participants [eg, financial assistance, healthcare, and prescription medications] requires Canadian citizenship), at least 19 years of age, who met criteria for homelessness or precarious housing, and current mental disorder status.^17^

Inclusion criteria specific to the current study included consent for researchers to access administrative data and linkable health records, and participant initiation of antipsychotic pharmacotherapy prior to study recruitment. In order to increase the probability of recruiting participants under current treatment, eligibility was limited to those with physician-diagnosed schizophrenia (International Classification of Diseases, Ninth Revision—Administrative [Medical Services Plan] records in British Columbia are based on ICD-9 codes, all diagnoses beginning with code 295), in the 10-year period prior to randomization. Previous studies support the validity of administrative data in relation to diagnoses of schizophrenia.^20,21^

### Interventions

Two Housing First interventions were compared to TAU. Both interventions satisfied formal fidelity assessments administered by an independent team led by staff from Pathways to Housing in New York.^22^

The SHF condition provided participants with a choice of single occupancy market-based apartments throughout the city of Vancouver, with home-based support from a psychiatrist-led, multidisciplinary ACT team available 24/7. Other than once-weekly meetings with the team, there were no requirements concerning treatment compliance or abstinence from substance use.

The CHF condition was implemented in a former hotel where participants had their own rooms, but without full kitchens. On-site, 24/7, multidisciplinary supports were provided by an existing agency with longstanding experience delivering harm reduction services (eg, needle exchange, supervised injection site, low-barrier housing, etc.) in Vancouver’s Downtown Eastside. CHF also provided recreational activities, communal meals, and vocational opportunities associated with daily operation of the facility (eg, laundry, cleaning). Front-desk staff continuously monitored the building and arrival/departure of guests to ensure the safety and security of residents. A medical examination room and pharmacy were also located on-site.

Individuals randomized to TAU did not receive housing supports and services through the study but were followed in the same manner as other study participants by members of the research team. The majority of participants randomized to TAU resided in the Downtown Eastside, a neighborhood well known for its concentration of health and social services targeted to the needs of homeless of people in Vancouver.^23,24^ These include housing units with varying levels of support, (eg, emergency shelters, single-room-occupancy hotels, purpose-built social housing units), meal programs, outreach services, HIV/AIDS and methadone treatment, drop-in centers, needle exchange, a supervised injection site, detox/recovery centers, and advocacy services.

All study participants, including those randomized to TAU, had free access to healthcare and all prescribed antipsychotic medication. Cost was not a barrier to pharmacological treatment. Research detailing components of the 3 treatment conditions has been published elsewhere.^17^

### Outcome Measures

#### Medication Possession Ratio.

Patient-specific medication details were drawn from a province-wide database (PharmaNet; see http://www2.gov.bc.ca/gov/content/health/health-drug-coverage/pharmacare-for-bc-residents/pharmanet) that records every prescription dispensed in British Columbia. PharmaNet has previously been shown to accurately reflect medication adherence for most patients.^25^

Expert consensus guidelines define acceptable adherence to antipsychotics as taking medication at least 80% of the time for which it is prescribed.^26^ We operationalized adherence using the medication possession ratio (MPR), which represents the percentage of time a given patient was dispensed prescribed medication (ie, number of days of medication supplied within refill interval divided by the total number of days in refill interval). The MPR is the preferred measure of adherence using administrative data^27^ and should be analyzed as a continuous variable^28^ to minimize bias.^29^ The MPR is also a valid correlate of important patient outcomes,^30^ including psychiatric hospital admission among patients with schizophrenia.^31,32^

The observation period we used to assess adherence to antipsychotic medication consisted of the length of time between the date of participant randomization (October 2009 to June 2011) and the study end date (March 31, 2013) or date of death. Accordingly, follow-up time varied between participants. MPR was calculated using number of days for which antipsychotics were prescribed during the follow-up period as the numerator, and the total postrandomization period (from randomization to study end date/death) as the denominator. Receipt of more than one antipsychotic prescription on any given day (antipsychotic polypharmacy) did not result in double counting or inflation of MPR (ie, upper bound = 1.0). Although participants may have received more than one antipsychotic agent over time, the current analysis did not focus on changes in medication regimen during the follow-up period.

Treatment guidelines recommend uninterrupted antipsychotic use up to 3 years following a first episode of psychosis, and indefinite prescription where clinically indicated.^33^ Because the current analysis selected individuals who had been diagnosed with schizophrenia prior to recruitment, and whose psychiatric symptoms were associated with long-term homelessness, we assumed that participants were in continuous need of their medications. This assumption conforms to the current standard of care of long-term antipsychotic use for maintenance therapy for schizophrenia,^34^ and to findings of a recently published review demonstrating that continuous antipsychotic use still outweighs the benefits of discontinuation.^35^

MPR in the prerandomization period was calculated using the same procedure as described above. A list of antipsychotic medications prescribed to participants in the pre- and postperiods is provided separately (see supplementary table 1).

#### Sociodemographic Variables.

Sociodemographic variables used in this study were collected via self-report at the baseline interview (prerandomization). These included: age (at recruitment and at first episode of homelessness), gender, ethnicity, education, marital status, chronic health conditions, blood-borne infectious diseases, and lifetime duration of homelessness. Age of participants was treated as a continuous variable. Ethnicity was collapsed into 3 groups: White, Aboriginal (including Inuit, Metis, First Nations Status, First Nations Non-Status, and Indigenous from outside Canada), and Other (including Asian, Black, Latin American, Indian-Caribbean, Middle Eastern, or other category). Education level was defined as highest level of formal education obtained at the time of recruitment and dichotomized into complete vs incomplete high school. Lifetime duration of homelessness was dichotomized using the median value and analyzed as a categorical variable.

### Sample Size Calculation

Examination of mental health services, including medications, was a secondary outcome analysis of the parent study, involving only those participants who had been diagnosed with schizophrenia and prescribed antipsychotics prior to recruitment. The parent study was powered to detect differences between groups on the primary outcome variable, housing stability.^36^ Previous studies using MPR as a measure of adherence to antipsychotic medications in comparable samples report a SD of approximately 0.29.^10,15^ For the current secondary analysis, we anticipated that a moderate effect size (Cohen’s *d* = 0.6, equivalent to a 17% difference) would be clinically meaningful. Based on this effect size with alpha level of .05, we estimated that 45 participants in each treatment arm would be sufficient to yield an 80% power to reject the null hypothesis of no effect in 2-sided statistical tests. We anticipated zero attrition due to the use of administrative data. No adjustment for multiplicity was made due to the exploratory nature of our outcome analysis.^37–41^

### Statistical Analysis

Continuous variables (eg, age and MPR) were presented using descriptive statistics (mean with SD or median with interquartile range as appropriate). Categorical variables (eg, gender and ethnic status) were presented using counts (*n*) and proportions (%). Independent sample *t* tests were used to compare continuous variables and Pearson chi-square test was used to compare categorical variables between groups. One-way ANOVA was used to compare MPR between the 3 conditions (SHF, CHF, and TAU). ANOVA is commonly used to analyze differences among group means and is considered a robust test against the normality assumption.^42^*P* values were reported based on Welch ANOVA, which is a well-established approach for performing ANOVA analysis when the homogeneity of variances assumption is not met and sample sizes are unequal.^43,44^

Following ANOVA, post hoc pairwise comparisons (SHF vs TAU and CHF vs TAU) were performed (Games-Howell test) to evaluate the intervention effect (ie, the difference in MPR between the intervention and TAU). Effect sizes are presented as relative differences with 95% CIs. Given there were no large differences in baseline characteristics between the 3 study groups, outcome analysis with adjustment of covariates was not performed.^45^ All reported *P* values are 2 sided.

As follow-up time varied between participants, sensitivity analysis was conducted using a standard follow-up time of 2 years across treatment conditions. Additional sensitivity analysis was performed in order to compare results based on physician diagnosis from administrative records to those based on diagnostic status derived from the Mini-international Neuropsychiatric Interview^46^ (administered at baseline). IBM SPSS Statistics 22.0^47^ was used to conduct all analyses.

## Results

### Sample Characteristics

**Fig. 1.**
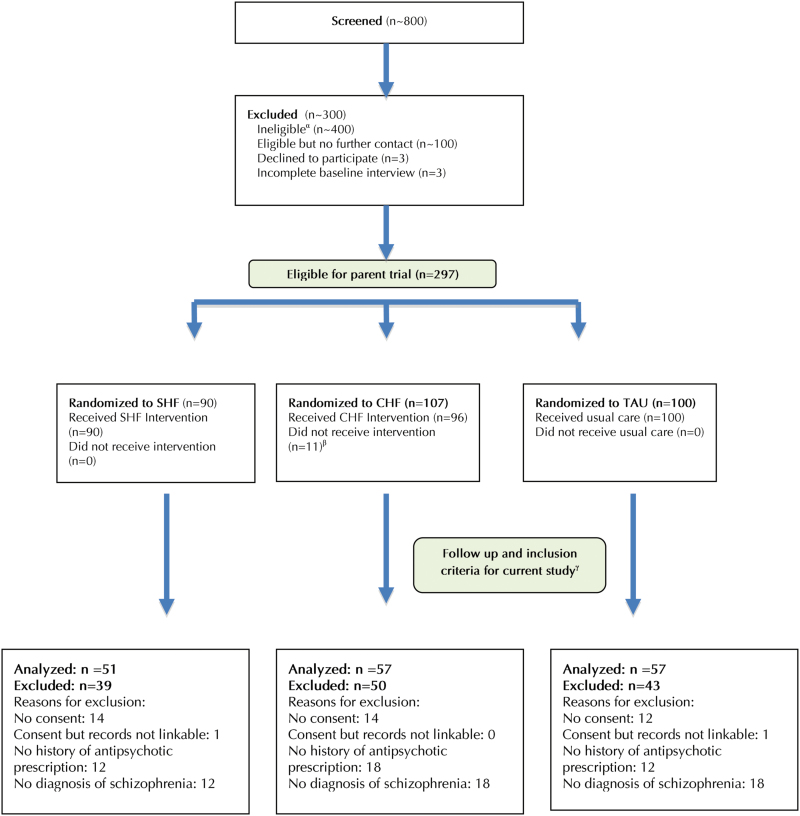
Participant flow through screening, assessment, and allocation to study interventions. ^α^Includes approximately 100 participants deemed ineligible via an informal telephone screen, 94 participants who were ineligible after formal in-person screening, and 200 participants who were eligible for a separate study that included participants with less severe needs. ^β^Includes 11 participants who could not be located after randomization or who left the study within 1 mo following randomization to intervention. ^γ^Eligibility criteria for the current study included participant consent to access of administrative health data, linkable health records, initiation of antipsychotic prescription prior to recruitment, and ICD-9 diagnosis of psychotic disorder. All eligible participants were followed to the study end date or death (no loss to follow-up). CHF, congregate Housing First; SHF, scattered-site Housing First; TAU, treatment as usual.

Of the 297 individuals recruited to the parent trial, 165 met eligibility criteria for the current study (figure 1). Among those randomized, 72% (*n* = 213) consented to access of administrative health data and had received at least one antipsychotic prescription prior to recruitment. Of these, 77% (*n* = 165) had a diagnosis of schizophrenia, comprising the current sample.

Participant baseline characteristics are shown in table 1. The eligible sample consisted predominantly of men (76%), with a mean age of 40 years, who identified ethnically as White (55%). Nearly half the sample (47%) reported a single episode of homelessness longer than 1 year. Mean age of first homelessness was 29 years. Eligible participants were significantly less likely than those excluded to have experienced cumulative lifetime homelessness of more than 3 years (42% vs 62%; *P* = .001). As shown in table 2, no significant differences were found between individuals randomized to CHF, SHF, and TAU, including prerandomization MPR (ranging from 0.44 to 0.48). Notably, scores from the SF-12 Health Summary and the Multnomah Community Ability scales demonstrate no significant differences in illness severity and level of impairment between treatment groups.

**Table 1.** Sociodemographic Characteristics of Vancouver At Home Participants at the Time of Recruitment (*n* = 297)

Variable	Entire Sample (*n* = 297)	Eligible Sample^a^ (*n* = 165)	Ineligible Sample^b^ (*n* = 132)	*P* Value^c^
	*n* (%)/Mean (SD)	*n* (%)/Mean (SD)	*n* (%)/Mean (SD)	
Age at recruitment (in years)	39.7 (11.2)	40.2 (11.1)	39.1 (11.4)	.406
Age of first homelessness (in years)	28.7 (12.5)	28.9 (12.6)	28.5 (12.5)	.822
Women	76 (26)	40 (24)	36 (28)	.521
Ethnicity				
Aboriginal	44 (15)	23 (14)	21 (16)	
White	170 (57)	91 (55)	79 (60)	
Other	83 (28)	51 (31)	32 (24)	.440
Incomplete high school	179 (61)	100 (61)	79 (60)	.855
Single/never married	214 (73)	121 (74)	93 (72)	.747
Lifetime duration of homelessness (>3 y)^d^	148 (51)	67 (42)	81 (62)	**.001**
Blood-borne infectious disease (HIV, hepatitis B or C)	87 (30)	43 (26)	44 (34)	.152
Chronic medical conditions (≥3)	189 (64)	100 (61)	89 (67)	.225

*Note*: Significant value of *P* <.05 is indicated in bold.

^a^Of 297 participants, 213 provided consent to access of administrative health data, initiated antipsychotic pharmacotherapy prior to recruitment, and were linkable to health records. Of these, 165 (77%) had a diagnosis of psychotic disorder and were included in the primary analysis.

^b^Of 132 participants, 40 did not provide consent to access of administrative data, 2 were not linkable to health records, 26 initiated antipsychotic pharmacotherapy in postrecruitment period, 16 had no history of antipsychotic prescription during the study period, and 48 had no diagnosis of psychotic disorder.

^c^ *P* values were based on comparison of characteristics between eligible participants and ineligible participants in the entire sample.

^d^Median value (36 mo) designated as the cutoff.

**Table 2.** Baseline Comparison of Sociodemographic and Related Characteristics of Eligible Participants by Study Arm (*n* = 165)

Variable	Congregate Site (*n* = 57)	Scattered Site (*n* = 51)	Treatment As Usual (*n* = 57)	*P* Value
	*n* (%)/Mean (SD)	*n* (%)/Mean (SD)	*n* (%)/Mean (SD)	
Age at recruitment (in years)	38.7 (11.1)	40.4 (11.0)	41.5 (11.1)	.396
Age of first homelessness (in years)	28.4 (12.4)	28.1 (12.1)	30.0 (13.4)	.713
Women	14 (25)	11 (22)	15 (27)	.821
Ethnicity				
Aboriginal	11 (19)	6 (12)	6 (11)	
White	30 (53)	28 (55)	33 (58)	
Other	16 (28)	17 (33)	18 (32)	.695
Incomplete high school	34 (61)	31 (62)	35 (61)	.991
Single/never married	44 (77)	34 (67)	43 (77)	.379
Lifetime duration of homelessness (>3 y)^a^	21 (38)	22 (46)	24 (43)	.680
Blood-borne infectious disease (HIV, hepatitis B or C)	17 (30)	10 (20)	16 (29)	.415
Chronic medical conditions (≥3)	33 (58)	27 (53)	40 (70)	.164
MCAS^b^ score	48.6 (7.1)	50.2 (6.1)	49.6 (6.5)	.418
SF-12 physical health^c^ score	47.8 (13.7)	48.4 (11.9)	44.5 (12.0)	.218
SF-12 mental health^d^ score	35.6 (15.5)	38.3 (13.1)	38.0 (13.0)	.535
Medication possession ratio in prerandomization period	0.47 (0.30)	0.48 (0.27)	0.44 (0.30)	.676
Initiation of antipsychotics in prerandomization period				
<5 y	17 (30)	18 (35)	17 (30)	
5–10 y	18 (32)	14 (27)	14 (25)	
>10 y	22 (39)	19 (37)	26 (46)	.845

*Note:* MCAS, Multnomah Community Ability Scale; SF, Short-Form Survey.

^a^Median value (36 mo) designated as the cutoff.

^b^Higher MCAS score indicates lower disability.

^c^Zero score indicates lowest level of health and 100 indicates highest level of health.

^d^Zero score indicates lowest level of health and 100 indicates highest level of health.

### Antipsychotic MPRs

Adherence to prescribed antipsychotics in the postrandomization period in each study arm is shown in table 3. Overall MPR was 0.64 (SD: 0.32) and approached the 0.80 threshold (0.78; SD: 0.21) among individuals randomized to SHF. MPRs in CHF and TAU were 0.61 (SD: 0.32) and 0.55 (SD: 0.37), respectively.

**Table 3.** Antipsychotic Medication Prescription Details in the Postrandomization Period (*n* = 165)

Variables	All (*n* = 165)	Congregate Site (*n* = 57)	Scattered Site (*n* = 51)	Treatment As Usual (*n* = 57)
Raw number of days with antipsychotic medication				
Mean (SD)	611.3 (346.4)	578.5 (347.4)	713.5 (253.4)	552.6 (399.2)
Median (IQR)	682 (340–907)	615 (291–862)	775 (540–887)	525 (224–945)
Follow-up time (days) between randomization and study end				
Mean (SD)	953.2 (217.1)	966.6 (205.7)	922 (229.8)	967.8 (217.6)
Median (IQR)	984 (880–1096)	983 (907–1080)	930 (765–1115)	1027 (913–1087)
Total follow-up time (person-years)	430.6	150.8	128.7	151.0
Number of days with antipsychotic medication (per person-year)	234.2	218.7	282.7	208.6
Medication possession ratio				
Mean (SD)	0.64 (0.32)	0.61 (0.32)	0.78 (0.21)	0.55 (0.37)
Median (IQR)	0.77 (0.38–0.92)	0.71 (0.39–0.89)	0.87 (0.71–0.93)	0.57 (0.27–0.92)
# of pharmacy encounters for antipsychotic medication (per person-year)	147.1	167.3	180.2	98.9

*Note:* IQR, interquartile range.

Median MPR in SHF was 0.87, meaning that adherence among half of participants in this condition far exceeded the 0.80 threshold. The skewness of the MPRs in both SHF and CHF implies that some participants responded strongly to Housing First, whereas a subgroup of participants did not. The number of antipsychotic-related pharmacy transactions was highest among participants randomized to SHF (180 per person-year), followed by those in CHF (167 per person-year), and lowest among those in the TAU group (99 per person-year).

Mean follow-up time for the entire cohort was 2.6 years. Eight participants died (see supplementary table 2 for cause of death) during the study period: 4 in SHF, 2 in CHF, and 2 in TAU. Under-ascertainment of death was unlikely, due to very high levels of retention in all study groups (including a 90% completion rate on prespecified in-person interviews) and consistent evidence of postrandomization receipt of health and social services as recorded in administrative data.

#### Housing First: Intervention Effects.

Welch ANOVA (see table 4) indicates that MPR was significantly different between study arms (*P* < .001)—because Levene’s test for homogeneity of variance was significant (*P* < .05), the overall *P* value was based on Welch ANOVA test. Post hoc comparisons demonstrate that SHF resulted in a significant intervention effect compared to TAU (relative difference: 0.24; 95% CI: 0.10–0.37, adjusted *P* < .001) while CHF was not significantly different from TAU (relative difference: 0.06; 95% CI: −0.10 to 0.21, adjusted *P* = .643).

**Table 4.** One-Way ANOVA Analysis Estimating Intervention Effects on Medication Possession Ratio in the Postrandomization Period (*n* = 165)

Study Arms	Medication Possession Ratio	*P* Value From ANOVA^a^	Intervention Effect	Adjusted *P* Value^b^ for Pairwise Comparisons
	Mean (95% CI)		Mean (95% CI)	
Based on administrative health data in last 10 y^c^				
Participants with psychotic disorder (*n* = 165)				
Congregate site (*n* = 57)	0.61 (0.52, 0.69)	<.001	0.06 (−0.10, 0.21)	.643
Scattered site (*n* = 51)	0.78 (0.73, 0.84)		0.24 (0.10, 0.37)	<.001
Treatment as usual (*n* = 57)	0.55 (0.45, 0.65)		Reference	Reference
Based on MINI diagnosis				
Participants with psychotic disorder (*n* = 154)				
Congregate site (*n* = 55)	0.58 (0.48, 0.67)	<.001	0.04 (−0.12, 0.20)	.816
Scattered site (*n* = 45)	0.79 (0.73, 0.85)		0.25 (0.12, 0.39)	<.001
Treatment as usual (*n* = 54)	0.54 (0.44, 0.64)		Reference	Reference

*Note:* MINI, Mini-International Neuropsychiatric Interview.

^a^Because Levene’s test for homogeneity of variance was significant (*P* < .05), the overall *P* value was based on Welch ANOVA test.

^b^Games-Howell test was used to adjust for family-wise errors between study arms.

^c^ICD-9 schizophrenic psychoses (codes 295.0–295.9).

Repeating the forgoing analyses after replacing physician diagnosis with diagnostic status derived from the Mini-international Neuropsychiatric Interview (administered at baseline) resulted in the same pattern of findings reported above (see table 4). Further sensitivity analysis using a standardized follow-up time of 2 years across treatment groups confirmed the above findings (see supplementary table 3).

## Discussion

Assignment to SHF produced a significant increase in adherence compared to TAU. Randomization to CHF was also associated with an increase in MPR, although the difference was not statistically significant. To our knowledge, this study is the first randomized controlled trial to examine the impact of housing interventions on adherence to antipsychotic medication in a sample of homeless adults with psychotic disorders.

Our results demonstrate that Housing First in market rental accommodations with ACT can effectively increase antipsychotic adherence to recommended levels among people who were homeless and diagnosed with schizophrenia. This finding adds to the demonstrated effectiveness of ACT with this population^14,48^ and supports the use of scattered-site housing as described by Pathways to Housing.^11^

Numerous studies have reported benefits of SHF for people with serious mental illness who experienced homelessness, including improved residential stability^49^ and reduced service costs.^50^ Previously reported findings from the Vancouver At Home study (the parent study for the current trial) have shown that, compared to TAU, participants assigned to SHF spent more days stably housed,^51^ experienced better community integration,^52^ and had significantly fewer criminal convictions^53^ and emergency department visits^54^ than those assigned to TAU. Similar differences were not observed between CHF and TAU, and these findings may be a function of clinical improvements that were specific to SHF.

Access to medications has been identified as a potentially important factor contributing to adherence to psychotropic prescriptions among homeless veterans.^55^ However, the CHF condition in the current trial (which included an on-site pharmacy) resulted in little evidence of superior antipsychotic adherence. The number of antipsychotic-related pharmacy transactions per person-year in CHF and SHF (167.3 and 180.2, respectively) were both higher than in TAU (98.9). The relatively high overall frequency of antipsychotic-related pharmacy transactions may have been medically advised to help ensure that clients neither lost nor forgot to take their medications. The fact that MPR was significantly higher in SHF suggests that improving adherence to antipsychotic medication in this population involves factors beyond access to medical resources. Important considerations include patient attitudes to medications, as well as clinical priorities and attitudes of prescribing physicians.

Our results demonstrate that usual services and supports available to homeless people with schizophrenia in Vancouver were associated with low rates of antipsychotic adherence. The observed MPR (0.55 in the postperiod) in TAU was substantially below consensus guidelines. Suboptimal adherence and consequent symptom persistence are associated with a range of adverse outcomes, including impaired functioning, self-neglect, and decreased quality of life,^56^ which may prevent access to services and supports needed to maintain health and overcome homelessness. These findings reinforce the importance of further expanding Housing First with ACT. They also underscore the need for immediate action to increase antipsychotic adherence among those who remain homeless while living with schizophrenia. One example of a potentially promising practice in this context is providing patients with financial incentives.^57,58^

Although self-report remains the most common method of assessing medication adherence,^59^ the predictive validity of subjective adherence measures is poor.^60^ Administrative data from a robust surveillance system, universal drug coverage, and no loss to follow-up are strengths of the current study. Nonetheless, some limitations should be considered.

MPR is an approximation of adherence behavior. Unlike directly observed therapy, MPR does not provide information on actual medication consumption. Use of pharmacy records may overestimate patient adherence, particularly as we did not account for hospitalization days in our analysis, although our use of a relatively long timeframe (>2.5 y) limits this potential for upward bias.^61^ Moreover, MPR does not differentiate between monotherapy and polypharmacy. This is potentially problematic, as antipsychotic polypharmacy is associated with refractory symptoms^62^ and greater illness severity.^63^

Circumstances associated with homelessness and street involvement (eg, lack of proper storage and routine, vulnerability to theft, etc.) may contribute to upward bias in MPR. In the current study, this potential would have been greatest among participants in TAU, as those in CHF and SHF were housed. Accordingly, upward bias of MPR in the current analysis likely contributed to even greater treatment effect.

Treatment interventions in SHF and CHF satisfied independent and detailed fidelity assessments, and support services in both groups were designed to be comparable in intensity and composition (ie, supports were delivered by multidisciplinary teams and available 24/7). However, it is possible that differences in clinical style arose and are unaccounted for in these analyses. Given their longstanding involvement with harm reduction services prior to this study, CHF support staff may have emphasized these principles (eg, greater tolerance for substance use/abuse).

The vast majority of participants were recruited from Vancouver’s Downtown Eastside. Individuals randomized to SHF were subject to norms established in existing market accommodations, where they could comprise at most 20% of the tenants in any single building.^17^ Those randomized to CHF were housed among other study participants and may have been more likely to carry forward norms derived from their previous neighborhood. This may have resulted in substantially different sociocultural norms and living environments between the 2 treatment conditions, with associated differences in resident behavior, motivation, and adherence.

We are not able to attribute differences in MPR to housing/support models alone. For instance, random assignment to a preferred treatment condition may promote treatment adherence and positive outcomes, while assignment to a less-preferred condition might limit motivation.^64^ Some evidence suggests that homeless consumers of mental health services—when given a choice—prefer market-based, independent tenancies without on-site services to congregate-style accommodations.^65^ However, other research found that individual housing preferences may have little influence on clinical outcomes, per se.^66^ Baseline participant preferences in our trial were not measured. The current findings contribute to the development of hypotheses for future trials. Research is needed to investigate the mechanisms that contribute to improvements in adherence and to examine the relationship between antipsychotic adherence and previously reported benefits of Housing First.

## Conclusions

Homeless participants with schizophrenia randomized to SHF exhibited significantly higher antipsychotic medication adherence—approaching guideline levels—than those in TAU. There were no significant differences between CHF and TAU, with both groups exhibiting very low adherence. Merely increasing access to medications, such as the provision of an on-site pharmacy, may not be sufficient to cause significant improvement in adherence. Further implementation of market-based housing with ACT is indicated as a means of increasing adherence to antipsychotic treatment among homeless people with schizophrenia.

## Supplementary Material

Supplementary data are available at *Schizophrenia Bulletin* online.

## Funding

This work was supported by the Mental Health Commission of Canada (2009s0231). S.N.R. received funding from the Canadian Institutes of Health Research (Banting & Best Doctoral Research Award 2013–2016) and the Imperial Order Daughters of the Empire (Doctoral Fellowship 2015–2016). S.F. received research funding from Wellcome Trust (095806).

## Supplementary Material

Supplementary Table 1

Supplementary Table 2

Supplementary Table 3

## References

[1] Fazel S Geddes JR Kushel M The health of homeless people in high-income countries: descriptive epidemiology, health consequences, and clinical and policy recommendations. Lancet. 2014;384:1529–1540. 25390578 10.1016/S0140-6736(14)61132-6 PMC4520328

[2] Solari C Cortes A Brown S Khadduri J Culhane DP The 2012 Annual Homelessness Assessment Report to Congress: Volume 2, Estimates of Homelessness in the United States https://works.bepress.com/dennis_culhane/135/ Published September 2013. Accessed May 7, 2016.

[3] Gaetz S Donaldson J Richter T Gulliver T. The State of Homelessness in Canada 2013. Toronto, Canada: Canadian Homelessness Research Network Press; 2013.

[4] Folsom D Jeste DV Schizophrenia in homeless persons: a systematic review of the literature. Acta Psychiatr Scand. 2002;105:404–413. 12059843 10.1034/j.1600-0447.2002.02209.x

[5] Chong MT Yamaki J Harwood M Assessing health conditions and medication use among the homeless community in Long Beach, California. J Res Pharm Pract. 2014;3:56–61. 25114938 10.4103/2279-042X.137073 PMC4124681

[6] Coe AB Moczygemba LR Gatewood SB Osborn RD Matzke GR Goode JV Medication adherence challenges among patients experiencing homelessness in a behavioral health clinic. Res Social Adm Pharm. 2015;11:e110–e120. 23218849 10.1016/j.sapharm.2012.11.004 PMC3733792

[7] Novick D Haro JM Suarez D Perez V Dittmann RW Haddad PM Predictors and clinical consequences of non-adherence with antipsychotic medication in the outpatient treatment of schizophrenia. Psychiatry Res. 2010;176:109–113. 20185182 10.1016/j.psychres.2009.05.004

[8] Ascher-Svanum H Faries DE Zhu B Ernst FR Swartz MS Swanson JW Medication adherence and long-term functional outcomes in the treatment of schizophrenia in usual care. J Clin Psychiatry. 2006;67:453–460. 16649833 10.4088/jcp.v67n0317

[9] Gilmer TP Dolder CR Lacro JP Adherence to treatment with antipsychotic medication and health care costs among Medicaid beneficiaries with schizophrenia. Am J Psychiatry. 2004;161:692–699. 15056516 10.1176/appi.ajp.161.4.692

[10] Rezansoff SN Moniruzzaman A Fazel S Procyshyn R Somers JM Adherence to antipsychotic medication among homeless adults in Vancouver, Canada: a 15-year retrospective study [published online ahead of print June 23, 2016]. Soc Psychiatry Psychiatr Epidemiol. doi:10.1007/s00127-016-1259-7. 10.1007/s00127-016-1259-7 PMC5091737 27338740

[11] Tsemberis S Eisenberg RF Pathways to housing: supported housing for street-dwelling homeless individuals with psychiatric disabilities. Psychiatr Serv. 2000;51:487–493. 10737824 10.1176/appi.ps.51.4.487

[12] Collins SE Malone DK Clifasefi SL Housing retention in single-site Housing First for chronically homeless individuals with severe alcohol problems. Am J Public Health. 2013;103(suppl 2):S269–S274. 24148063 10.2105/AJPH.2013.301312 PMC3969126

[13] Manuel JI Covell NH Jackson CT Essock SM Does Assertive Community Treatment increase medication adherence for people with co-occurring psychotic and substance use disorders? J Am Psychiatr Nurses Assoc. 2011;17:51–56. 21659294 10.1177/1078390310395586 PMC4164343

[14] Dixon L Weiden P Torres M Lehman A Assertive Community Treatment and medication compliance in the homeless mentally ill. Am J Psychiatry. 1997;154:1302–1304. 9286194 10.1176/ajp.154.9.1302

[15] Valenstein M McCarthy JF Ganoczy D Assertive Community Treatment in veterans affairs settings: impact on adherence to antipsychotic medication. Psychiatr Serv. 2013;64:445–451. 23412131 10.1176/appi.ps.201100543

[16] McHugo GJ Bebout RR Harris M A randomized controlled trial of integrated versus parallel housing services for homeless adults with severe mental illness. Schizophr Bull. 2004;30:969–982. 15957201 10.1093/oxfordjournals.schbul.a007146

[17] Somers JM Patterson ML Moniruzzaman A Vancouver At Home: pragmatic randomized trials investigating Housing First for homeless and mentally ill adults. Trials. 2013;14:365. 24176253 10.1186/1745-6215-14-365 PMC4228396

[18] Barker S Barron N McFarland BH Bigelow DA Carnahan T A Community Ability Scale for chronically mentally ill consumers: Part II. Applications. Community Ment Health J. 1994;30:459–472. 7851100 10.1007/BF02189063

[19] Ware J Jr Kosinski M Keller SD A 12-Item Short-Form Health Survey: construction of scales and preliminary tests of reliability and validity. Med Care. 1996;34:220–233. 8628042 10.1097/00005650-199603000-00003

[20] Ekholm B Ekholm A Adolfsson R Evaluation of diagnostic procedures in Swedish patients with schizophrenia and related psychoses. Nord J Psychiatry. 2005;59:457–464. 16316898 10.1080/08039480500360906

[21] Marrie RA Fisk JD Yu BN; CIHR Team in the Epidemiology and Impact of Comorbidity on Multiple Sclerosis Mental comorbidity and multiple sclerosis: validating administrative data to support population-based surveillance. BMC Neurol. 2013;13:16. 23388102 10.1186/1471-2377-13-16 PMC3599013

[22] Tsemberis SJ. Housing First: The Pathways Model to End Homelessness for People With Mental Illness and Addiction. Center City, MN: Hazeldon; 2010.

[23] Campbell L Boyd N Culbert L. A Thousand Dreams: Vancouver’s Downtown Eastside and the Fight of Its Future. Vancouver, Canada: Greystone Books; 2010.

[24] Somers JM Rezansoff SN Moniruzzaman A Zabarauckas C High-frequency use of corrections, health, and social services, and association with mental illness and substance use. Emerg Themes Epidemiol. 2015;12:17. 26691347 10.1186/s12982-015-0040-9 PMC4683696

[25] Dahri K Shalansky SJ Jang L Jung L Ignaszewski AP Clark C Accuracy of a provincial prescription database for assessing medication adherence in heart failure patients. Ann Pharmacother. 2008;42:361–367. 18303147 10.1345/aph.1K385

[26] Velligan DI Weiden PJ Sajatovic M; Expert Consensus Panel on Adherence Problems in Serious and Persistent Mental Illness The expert consensus guideline series: adherence problems in patients with serious and persistent mental illness. J Clin Psychiatry. 2009;70(suppl 4):1–46; quiz 47. 19686636

[27] Hess LM Raebel MA Conner DA Malone DC Measurement of adherence in pharmacy administrative databases: a proposal for standard definitions and preferred measures. Ann Pharmacother. 2006;40:1280–1288. 16868217 10.1345/aph.1H018

[28] Andrade SE Kahler KH Frech F Chan KA Methods for evaluation of medication adherence and persistence using automated databases. Pharmacoepidemiol Drug Saf. 2006;15:565–574; discussion 575. 16514590 10.1002/pds.1230

[29] Tueller JT Deboeck PR VanDorn RA Getting less of what you want: reductions in statistical power and increased bias when categorizing medication adherence data [published online ahead of print February 27, 2016]. J Behav Med. 2016. doi:10.1007/s10865-016-9727-9. 10.1007/s10865-016-9727-9 PMC5002256 26921166

[30] Steiner JF Koepsell TD Fihn SD Inui TS A general method of compliance assessment using centralized pharmacy records. Description and validation. Med Care. 1988;26:814–823. 3398608 10.1097/00005650-198808000-00007

[31] Valenstein M Blow FC Copeland LA Poor antipsychotic adherence among patients with schizophrenia: medication and patient factors. Schizophr Bull. 2004;30:255–264. 15279044 10.1093/oxfordjournals.schbul.a007076

[32] Weiden PJ Kozma C Grogg A Locklear J Partial compliance and risk of rehospitalization among California Medicaid patients with schizophrenia. Psychiatr Serv. 2004;55:886–891. 15292538 10.1176/appi.ps.55.8.886

[33] National Institute for Health and Care Excellence (NICE). Psychosis and Schizophrenia in Adults: Prevention and Management https://www.nice.org.uk/guidance/cg178 Published February 2014. Accessed May 2, 2016. 32207892

[34] Harrow M Jobe TH Faull RN Do all schizophrenia patients need antipsychotic treatment continuously throughout their lifetime? A 20-year longitudinal study. Psychol Med. 2012;42:2145–2155. 22340278 10.1017/S0033291712000220

[35] Emsley R Kilian S Phahladira L How long should antipsychotic treatment be continued after a single episode of schizophrenia? Curr Opin Psychiatry. 2016;29:224–229. 26967313 10.1097/YCO.0000000000000249

[36] Goering PN Streiner DL Adair C The At Home/Chez Soi trial protocol: a pragmatic, multi-site, randomised controlled trial of a Housing First intervention for homeless individuals with mental illness in five Canadian cities. BMJ Open. 2011;1:e000323. 10.1136/bmjopen-2011-000323 PMC3221290 22102645

[37] Wason JM Stecher L Mander AP Correcting for multiple-testing in multi-arm trials: is it necessary and is it done? Trials. 2014;15:364. 25230772 10.1186/1745-6215-15-364 PMC4177585

[38] Baron G Perrodeau E Boutron I Ravaud P Reporting of analyses from randomized controlled trials with multiple arms: a systematic review. BMC Med. 2013;11:84. 23531230 10.1186/1741-7015-11-84 PMC3621416

[39] Freidlin B Korn EL Gray R Martin A Multi-arm clinical trials of new agents: some design considerations. Clin Cancer Res. 2008;14:4368–4371. 18628449 10.1158/1078-0432.CCR-08-0325

[40] Proschan MA Waclawiw MA Practical guidelines for multiplicity adjustment in clinical trials. Control Clin Trials. 2000;21:527–539. 11146147 10.1016/s0197-2456(00)00106-9

[41] Rothman KJ No adjustments are needed for multiple comparisons. Epidemiology. 1990;1:43–46. 2081237

[42] Kirk RE. Experimental Design: Procedures for the Behavioral Sciences. Pacific Grove, CA: Brooks/Cole; 1995.

[43] Brown MB Forsythe AB The small sample behavior of some statistics which test the equality of several means. Technometrics. 1974;16:129–132.

[44] Welch BL On the comparison of several mean values; an alternative approach. Biometrika. 1951;38:330–336.

[45] Senn S Testing for baseline balance in clinical trials. Stat Med. 1994;13:1715–1726. 7997705 10.1002/sim.4780131703

[46] Sheehan DV Lecrubier Y Sheehan KH The Mini-International Neuropsychiatric Interview (M.I.N.I.): the development and validation of a structured diagnostic psychiatric interview for DSM-IV and ICD-10. J Clin Psychiatry. 1998;59(suppl 20):22–33; quiz 34. 9881538

[47] Corp IBM. Released. IBM SPSS Statistics for Windows, Version 22.0. Armonk, NY: IBM Corp; 2013.

[48] Coldwell CM Bender WS The effectiveness of Assertive Community Treatment for homeless populations with severe mental illness: a meta-analysis. Am J Psychiatry. 2007;164:393–399. 17329462 10.1176/ajp.2007.164.3.393

[49] Stefancic A Tsemberis S Housing First for long-term shelter dwellers with psychiatric disabilities in a suburban county: a four-year study of housing access and retention. J Prim Prev. 2007;28:265–279. 17592778 10.1007/s10935-007-0093-9

[50] Rosenheck R Kasprow W Frisman L Liu-Mares W Cost-effectiveness of supported housing for homeless persons with mental illness. Arch Gen Psychiatry. 2003;60:940–951. 12963676 10.1001/archpsyc.60.9.940

[51] Palepu A Patterson ML Moniruzzaman A Frankish CJ Somers J Housing First improves residential stability in homeless adults with concurrent substance dependence and mental disorders. Am J Public Health. 2013;103(suppl 2):e30–e36. 10.2105/AJPH.2013.301628 PMC3969112 24148035

[52] Patterson ML Moniruzzaman A Somers JM Community participation and belonging among formerly homeless adults with mental illness after 12 months of Housing First in Vancouver, British Columbia: a randomized controlled trial. Community Ment Health J. 2014;50:604–611. 24323139 10.1007/s10597-013-9672-9

[53] Somers JM Rezansoff SN Moniruzzaman A Palepu A Patterson M Housing First reduces re-offending among formerly homeless adults with mental disorders: results of a randomized controlled trial. PLoS ONE. 2013;8:e72946. 24023796 10.1371/journal.pone.0072946 PMC3762899

[54] Russolillo A Somers JM McCandless L Patterson M Moniruzzaman A Emergency department utilization among formerly homeless adults with mental disorders after 1-year of Housing First: a randomized controlled trial. Int J Housing Policy 2014;14:79–97.

[55] Hermes E Rosenheck R Psychopharmacologic services for homeless veterans: comparing psychotropic prescription fills among homeless and non-homeless veterans with serious mental illness. Community Ment Health J. 2016;52:142–147. 26202545 10.1007/s10597-015-9904-2

[56] Haddad PM Brain C Scott J Nonadherence with antipsychotic medication in schizophrenia: challenges and management strategies. Patient Relat Outcome Meas. 2014;5:43–62. 25061342 10.2147/PROM.S42735 PMC4085309

[57] Highton-Williamson E Barnicot K Kareem T Priebe S Offering financial incentives to increase adherence to antipsychotic medication. J Clin Psychopharmacol 2015;35:120–127. 25692797 10.1097/JCP.0000000000000276 PMC4352071

[58] Petry NM Rash CJ Byrne S Ashraf S White WB Financial reinforcers for improving medication adherence: findings from a meta-analysis. Am J Med. 2012;125:888–896. 22800876 10.1016/j.amjmed.2012.01.003 PMC3432682

[59] Velligan DI Weiden PJ Sajatovic M Assessment of adherence problems in patients with serious and persistent mental illness: recommendations from the Expert Consensus Guidelines. J Psychiatr Pract. 2010;16:34–45. 20098229 10.1097/01.pra.0000367776.96012.ca

[60] Kikkert MJ Koeter MW Dekker JJ The predictive validity of subjective adherence measures in patients with schizophrenia. Int J Methods Psychiatr Res. 2011;20:73–81. 21557378 10.1002/mpr.335 PMC6878246

[61] Vollmer WM Xu M Feldstein A Smith D Waterbury A Rand C Comparison of pharmacy-based measures of medication adherence. BMC Health Serv Res. 2012;12:155. 22691240 10.1186/1472-6963-12-155 PMC3413584

[62] Sernyak MJ Rosenheck R Clinicians’ reasons for antipsychotic coprescribing. J Clin Psychiatry. 2004;65:1597–1600. 15641863 10.4088/jcp.v65n1203

[63] Gallego JA Bonetti J Zhang J Kane JM Correll CU Prevalence and correlates of antipsychotic polypharmacy: a systematic review and meta-regression of global and regional trends from the 1970s to 2009. Schizophr Res. 2012;138:18–28. 22534420 10.1016/j.schres.2012.03.018 PMC3382997

[64] Macias C Gold PB Hargreaves WA Preference in random assignment: implications for the interpretation of randomized trials. Adm Policy Ment Health. 2009;36:331–342. 19434489 10.1007/s10488-009-0224-0 PMC2796239

[65] Schutt RK Goldfinger SM Housing preferences and perceptions of health and functioning among homeless mentally ill persons. Psychiatr Serv. 1996;47:381–386. 8689368 10.1176/ps.47.4.381

[66] O’Connell M Rosenheck R Kasprow W Frisman L An examination of fulfilled housing preferences and quality of life among homeless persons with mental illness and/or substance use disorders. J Behav Health Serv Res. 2006;33:354–365. 16752112 10.1007/s11414-006-9029-z

