# Supplementary material for: Housing First Improves Adherence to Antipsychotic Medication Among Formerly Homeless Adults With Schizophrenia: Results of a Randomized Controlled Trial
Source: Schizophr Bull. 2016 Sep 24;43(4):852–61. doi: 10.1093/schbul/sbw136 (PMC5274537; doi:10.1093/schbul/sbw136)
Supplement: Supplementary file 1 [file sbw136_suppl_Supplementary_Table_1.doc]

**Supplementary Table 1: Generic names of antipsychotic drugs prescribed to Vancouver At Home (VAH) participants during pre and post-randomization periods**

| **Generic name** | **% of total pharmacy encounters** |
| --- | --- |
| ARIPIPRAZOLE | 2.5 |
| CHLORPROMAZINE HCL | 0.5 |
| CLOZAPINE | 8.7 |
| FLUPENTIXOL DECANOATE | 0.7 |
| FLUPENTIXOL HCL | 0.9 |
| FLUPHENAZINE DECANOATE | 0.3 |
| FLUPHENAZINE HCL | 0 |
| HALOPERIDOL | 0.9 |
| HALOPERIDOL DECANOATE | 0.1 |
| HALOPERIDOL LACTATE | 0 |
| LOXAPINE HCL | 0 |
| LOXAPINE SUCCINATE | 7.3 |
| METHOTRIMEPRAZINE HCL | 0 |
| METHOTRIMEPRAZINE MALEATE | 1.2 |
| OLANZAPINE | 24.7 |
| PALIPERIDONE | 0 |
| PALIPERIDONE PALMITATE | 0.2 |
| PERPHENAZINE | 0 |
| PIMOZIDE | 0 |
| PIPOTIAZINE PALMITATE | 0.1 |
| QUETIAPINE FUMARATE | 33.9 |
| RISPERIDONE | 13.4 |
| RISPERIDONE MICROSPHERES | 1.5 |
| THIORIDAZINE HCL | 0 |
| TRIFLUOPERAZINE HCL | 0.3 |
| ZIPRASIDONE HCL | 1.8 |
| ZUCLOPENTHIXOL ACETATE | 0 |
| ZUCLOPENTHIXOL DECANOATE | 0.7 |
| ZUCLOPENTHIXOL HCL | 0.3 |
