# Supplementary material for: Housing First Improves Adherence to Antipsychotic Medication Among Formerly Homeless Adults With Schizophrenia: Results of a Randomized Controlled Trial
Source: Schizophr Bull. 2016 Sep 24;43(4):852–61. doi: 10.1093/schbul/sbw136 (PMC5274537; doi:10.1093/schbul/sbw136)
Supplement: Supplementary file 2 [file sbw136_suppl_Supplementary_Table_2.doc]

**Supplementary Table 2: Deaths among eligible participants in the post-randomization period (n=165).**

|  | **Death^[1]^** | **Total Person-Years^[2]^ (PYs)** | **Death per 100 PYs** | ***P* value^[3]^** |
| --- | --- | --- | --- | --- |
| **Overall (n=165)** | 8 | 430.6 | 1.9 |  |
| Congregate site (n=57)  Scattered site (n=51)  Treatment as usual (n=57) | 2  4  2 | 150.8  128.7  151.0 | 1.3  3.1  1.3 | 0.511 |

1. - Cause of death (based on ICD-10 codes) was confirmed from vital statistics for 6 of the 8 deaths as follows:
   2 - alcohol overdose; 1 - suicide; 1 – respiratory disease; 2 – not specified. The remaining 2 deaths were not confirmed by vital statistics, and cause of death cannot be substantiated using administrative data.
2. - Censoring date: March 31, 2013; date of death for deceased.
3. - Log Rank (Mantel-Cox) was used to compare the survival curve between study arms.
