# Supplementary material for: Housing First Improves Adherence to Antipsychotic Medication Among Formerly Homeless Adults With Schizophrenia: Results of a Randomized Controlled Trial
Source: Schizophr Bull. 2016 Sep 24;43(4):852–61. doi: 10.1093/schbul/sbw136 (PMC5274537; doi:10.1093/schbul/sbw136)
Supplement: Supplementary file 3 [file sbw136_suppl_Supplementary_Table_3.doc]

**Supplementary Table 3: Sensitivity analysis^[1]^ of intervention effects on medication possession ratio in the post-randomization period using a standardized 2-year follow-up (n=147).**

| **Study arms** | **Medication possession ratio**  **Mean (95% confidence interval)** | **P value from ANOVA^[2]^** | **Intervention effect**  **Mean (95% confidence interval)** | **Adjusted P value^[3]^ for pairwise comparisons** |
| --- | --- | --- | --- | --- |
| **(Based on administrative health data in last 10 years)^[4]^** |  |  |  |  |
| **Participants with psychotic disorder (n=147)**  Congregate site (n=51)  Scattered site (n=42)  Treatment as usual (n=54) | 0.61 (0.51, 0.70)  0.76 (0.70, 0.83)  0.57 (0.46, 0.67) | **0.002** | 0.04 (-0.13, 0.21)  0.20 (0.05, 0.34)  Reference | 0.834  **0.006**  Reference |

1. -Restricted to participants with at least two years of follow-up. MPR was re-calculated using number of days with antipsychotic agents in two years of follow up as the numerator and 730 days as the denominator.
2. As Levene’s test for homogeneity of variance was significant (p <0.05), the overall *P* value was based on Welch ANOVA test.
3. Games-Howell test was used to adjust for family-wise errors between study arms.
4. ICD-9 schizophrenic disorders (codes 295.0 – 295.9)
